# Homeostatic Imbalance in Epithelial Ducts and Its Role in Carcinogenesis

**DOI:** 10.6064/2012/132978

**Published:** 2012-07-12

**Authors:** Katarzyna A. Rejniak

**Affiliations:** ^1^Depatment of Integrated Mathematical Oncology, H. Lee Moffitt Cancer Center and Research Institute, 12902 Magnolia Drive, Room SRB-4 24000G, Tampa, FL 33612, USA; ^2^Department of Oncologic Sciences, College of Medicine, University of South Florida, Tampa, FL 33612, USA

## Abstract

An epithelial duct is a well-defined multicellular structure composed of tightly packed cells separating and protecting body compartments that are used for enzyme secretion and its transport across the internal. The structural and functional integrity (homeostasis) of such ducts is vital in carrying many life functions (breathing, lactation, production of hormones). However, the processes involved in maintaining the homeostatic balance are not yet fully understood. On the other hand, the loss of epithelial tissue architecture, such as filled lumens or ductal disorganization, are among the first symptoms of the emerging epithelial tumors (carcinomas). Using the previously developed biomechanical model of epithelial ducts: *IBCell*, we investigated how different signals and mechanical stimuli imposed on individual epithelial cells can impact the homeostatic (im)balance and integrity of the whole epithelial tissue. We provide a link between erroneous responses of individual epithelial cells to specific signals and the emerging ductal morphologies characteristic for preinvasive cancers observed in pathology specimens, or characteristic for multicellular structures arising from mutated cells cultured *in vitro*. We summarize our finding in terms of altered properties of epithelial cell polarization, and discuss the relative importance of various polarization signals on the formation of tumor-like multicellular structures.

## 1. Introduction 

Epithelia are one of the most abundant tissues in the human body and cover all cavities and surfaces of many organs, including the bronchi and alveoli of the lungs, breast ducts and lobules, gastrointestinal crypts, reproductive urinary tracts, and the endocrine glands. In their mature form, the simple epithelia (in contrast to stratified, multilayered tissues) are organized into monolayer sheets of tightly packed polarized cells that enclose lumens (either empty or filled with milk, semen, or air) to prevent leakage of molecules from one side of the epithelia to the other. The overall structure and properties of these well-organized multicellular systems emerge from physical interactions and chemical signals shared locally between individual neighboring cells. However, the processes involved in keeping the epithelial tissue integrity over the span of human lifetime are not yet fully understood. 

On one hand, certain control mechanisms are required that ensure tissue stabilization (e.g., via cell differentiation). On the other hand, some degree of adaptability to a range of conditions (e.g., stress or damage) is needed. All critical decisions regulating epithelial tissue integrity, such as initiation of cell growth, selection of the axis of cell division, or the induction of cell death, may be influenced by actions of neighboring cells and interactions between the cells and their dynamically evolving microenvironment. This includes diverse set of forces, such as compressive, tensile, or hydrostatic pressures, each of which plays a part in shaping the tissue [1–3]. The way in which cells respond to forces is dictated by their physical properties. In fact, cells are both elastic and viscous, meaning that they deform in a time-dependent manner upon applied forces and can return towards their initial form following removal of the stresses. Cells can also exert forces on their neighbors. Epithelial cells develop specialized cell-cell connections, such as adherens junctions, that mechanically attach cells to one another. Similarly, cell surface receptors, such as integrins, transmit forces from the external environment. Additionally, cells develop gap junctions that mediate the passage of chemical and electrical signals between neighboring cells and provide a mechanism for coordinating the activities of individual cells in the tissue. Thus, mechanical signals sensed by the cell result in activation of intracellular biochemical signaling pathways (the process called mechanotransduction), that in turn regulate cell behavior [4, 5]. 

This complex interplay allows for the emergence of more robust multicellular systems that can tolerate both external and internal perturbations [6]. Normal epithelial cells can adapt to certain perturbations in biochemical, genetic, and physical cues sensed from their immediate microenvironment. However, there are limitations to this plasticity, and when cell responses are compromised, they may induce a malignant behavior, such as filling the hollow ductal lumen with mutated cells and breaking through the basement membrane, that results in loss of tissue homeostasis. For example, early preinvasive forms of epithelial cancers, such as breast ductal carcinoma *in situ* (DCIS) or prostatic intraepithelial neoplasia (PIN), are both characterized by filled ductal lumens and ductal epithelium disorganization. Thus, the disruption of normal epithelial tissue architecture is one of the initial symptoms of progression to epithelial tumors [7]. It has been shown that changes in extracellular matrix structure, its stiffness, and mechanics can promote or suppress formation of malignant multicellular spheroids in 3D cell cultures [8, 9]. The physical forces and differences in cellular mechanics provide essential cues driving cell differentiation, growth, or death. In these cases, biomechanics underlies the abilities of the cell as well as the tissue to carry out normal function in health or to malfunction in disease. From this perspective, the emergence of preinvasive cancers can be viewed as tissue homeostatic imbalance in which the natural symbiosis between cellular and microenvironmental components is perturbed.

We previously developed a biomechanical computational model of deformable cells, *IBCell* (the Immersed Boundary model of a Cell, see appendix), that was used to test various cell-cell and cell-microenvironment interactions in the self-organizing polarized cysts. Here, we extend this mathematical framework to include alterations in mechanical stimuli responsible for cell epithelial polarization to investigate how this pressure imbalance impacts the overall tissue integrity. 

## 2. Results

**Figure 1:**
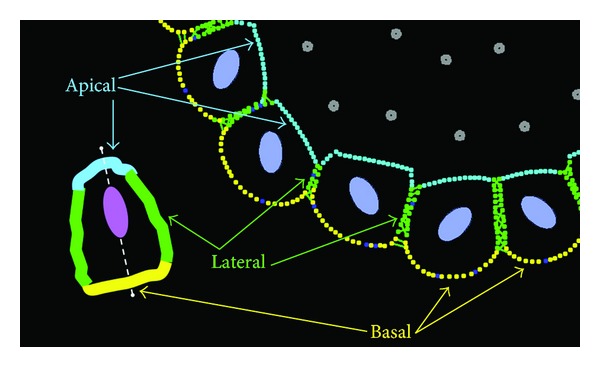
Schematics of the *IBCell* polarized epithelium. An epithelially polarized cell contains three membrane domains: lateral (green) in contact with other cells in the epithelium, basal (yellow) in contact with the extracellular matrix (ECM), and lateral (cyan) in contact with the lumen. These membrane domains in *IBCell *are composed of corresponding boundary points (receptors). *IBCell* contains also growth (blue) and death (grey) receptors. Colors of cell nuclei: viable cells (purple) and dying cells (red). Inset: a graphical representation of a polarized cell and its three different membrane domains.

Tissue homeostatic balance emerges from the integration of multiple subcellular, intercellular, extracellular, chemical, and physical signals and constraints. Understanding how this normal form and function of the tissues is maintained is vital for recognizing which elements play the key roles during transition from normal epithelia to preinvasive tumors. We previously developed a biomechanical model, *IBCell*, of the formation of normal epithelial acini (the 3D *in vitro* cultures resembling hollow epithelial cysts) and discussed both the necessary and sufficient conditions for their self-organization [10, 11]. In this approach, each epithelial cell is modeled as a deformable body with the fluid-like cytoplasm and an elastic membrane, equipped with a set of discrete receptors/sensors distributed along the cell boundary. Five types of receptors are considered here: growth, death, apical, lateral, and basal (see Figure 1), and, based on the configuration of its receptors, the cell undergoes one of several possible life processes: growth, division, apoptotic death, or epithelial polarization. For instance, the cell requires that a certain number of growth or death receptors to be active in order to initiate the process of growth or death, respectively. The *IBCell* receptors are meant to resemble the biological role of cell membrane receptors; however, some simplifications need to be introduced in the computational model. The biological receptors are highly dynamic and can be relocated or created in response to extracellular signals. The *IBCell *receptors are immobile, but their role can be modified (activated) based on local cues. The default state of all *IBCell* receptors is *growth*, but their status will be changed to *lateral*, if two cells are close to one another and develop the cell-cell adhesion link. The receptor's role will change to *basal*, if the level of the extracellular matrix (ECM) proteins in its vicinity is high enough to provide cell-ECM adhesion. The receptor will be activated as *apical* when the epithelial cell becomes polarized and develops the apical membrane domain. Finally, the receptor's status will be changed to *death* when the cell loses its adhesive connection with polarized cells. A particular cell life process will be initiated depending on whether the number of active receptors reaches a predefined threshold. These thresholds define cell intrinsic sensitivity to extrinsic signals. 

The fundamental feature of tissue epithelial architecture a well-defined apical-basolateral polarity of all individual cells (defined as asymmetrical distribution of cell components, especially membrane proteins). The membrane of epithelial cells is composed of noncoalescent apical and basolateral domains—with the apical side facing the lumen, the lateral sides adhering to other cells, and the basal side attached to the underlying basement membrane. In *IBCell,* epithelial polarity is acquired by developing three distinct membrane compartments (Figure 1): basal, defined by cell membrane receptors contacting the external media (red); lateral, defined by cell receptors being in contact with other cells (green); apical, composed of markers facing the hollow lumen (cyan). Depending on the tissue of origin, the hollow lumen may be a result of cell death (MCF10A breast cells [12, 13]) or vacuolar exocytosis (MDCK kidney cells [14]). Cell apoptotic death is modeled in *IBCell* by placing point sinks and sources along the membrane of the whole cell to release fluid from the cell interior to the extracellular space. In contrast, cell growth is modeled by locating point sources and sinks of fluid in such a way to increase the amount of fluid inside the cell (see the appendix for more details). Critical decisions regulating epithelial tissue integrity, such as cell growth, division, or death, can be triggered by signals from the neighboring cells and from the microenvironment. We will illustrate here how changes in cellular responses to such cues sensed by the host cells via their different membrane receptors may result in the disruption of epithelial tissue morphology. Specifically, we will use *IBCell* to examine how epithelial morphologies are changing as a result of disrupted or altered cellular responses to different constrains arising from cell epithelial polarization, such as ECM, apical, lateral, and death signals controlling cell mitosis, adhesion, or death.

### 2.1. Development of Normal Epithelial Cysts

**Figure 2:**
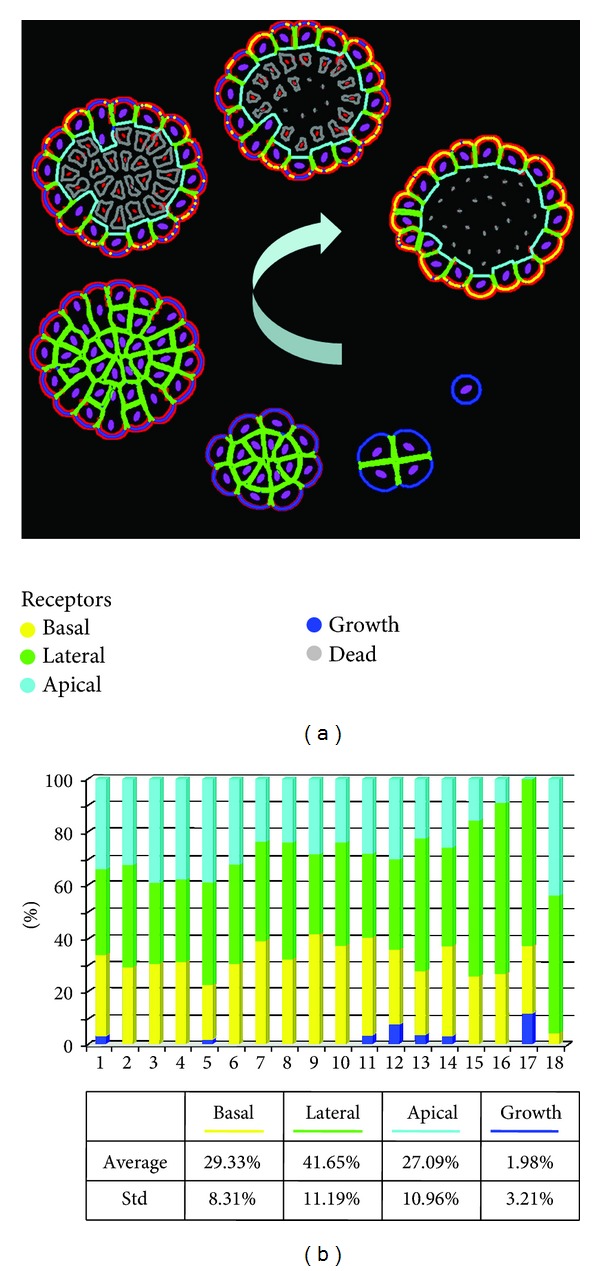
Emergence of polarized cells during normal epithelium development. (a) Sequence of consecutive stages in the development of normal epithelium from a single cell to a one layer of polarized cells enclosing the hollow lumen. (b). Distributions of membrane receptors in each polarized cell forming the final one-layered epithelial structure. Cell membrane receptors are color-coded identically in (a) and (b). The red bands surrounding the multicellular structures in (a) represent accumulation of ECM proteins.

Polarized cells forming the epithelial tissue are of cubical or columnar shape with three well-defined membrane domains each with specific proteins and lipids. However, the relative proportions of surface areas of these membrane sides are not known, and it is not known how changes in distribution of membrane-specific proteins can influence the epithelial development and maintenance. In order to identify the ranges of apical, lateral, and basal receptors in normally formed epithelial cells, we simulated the development of several self-organized epithelial cysts. One realization of our model is shown in Figure 2. The presented developmental stages are consistent with 3D *in vitro* cultures derived from nontumorigenic epithelial cell lines, for example, breast cells MCF10A that form hollow mammospheres [15, 16], prostate cells RWPE-1 that produce one-layered acini [17], or ovarian OVCAR-5 cells growing in a cyst-like form [18]. The acinar structure is formed from a single cell that upon consecutive divisions gives rise to an aggregate of randomly oriented cells consisting of two cell populations: outer cells having contact with the external medium and inner cells surrounded entirely by other cells. Subsequently, cells in the outer layer develop an apical-basal polarity and form a monolayer of epithelial cells. This is followed by apoptotic death of inner cells, resulting in the formation of the hollow lumen and in stabilization of the epithelial structure (Figure 2(a)). All final epithelial cells have been analyzed in order to quantify the percentage of various cell membrane receptors that guarantee proper cell polarization in the model (Figure 2(b)). 

On average, in our simulations of polarized epithelial ducts, each polarized cell contains about 30% of ECM receptors forming the basal membrane domain that is in contact with the external medium (such as matrigel *in vitro* or the extracellular matrix *in vivo*). Lateral domains composed of cell-cell adhesive receptors (such as cadherins or tight junctions) contain about half of all receptors and are divided between lateral sides being in contact with two adjacent cells. Most of the final epithelial cells in *IBCell* are of cuboidal shape; thus, their apical sides are composed of quite large numbers of apical markers (~25%). The active growth receptors in the final stable configurations are very rare (about 2% per cell, and many cells have no active growth receptors at all) in contrast to earlier stages when active growth receptors (blue) are dominant over other kinds of receptors. These values have been used in subsequent simulations for testing how changes in a particular signaling mechanism (from ECM, other cells, and lumen) lead to morphological alternations in epithelial structures.

### 2.2. Alterations in ECM Signals

**Figure 3:**
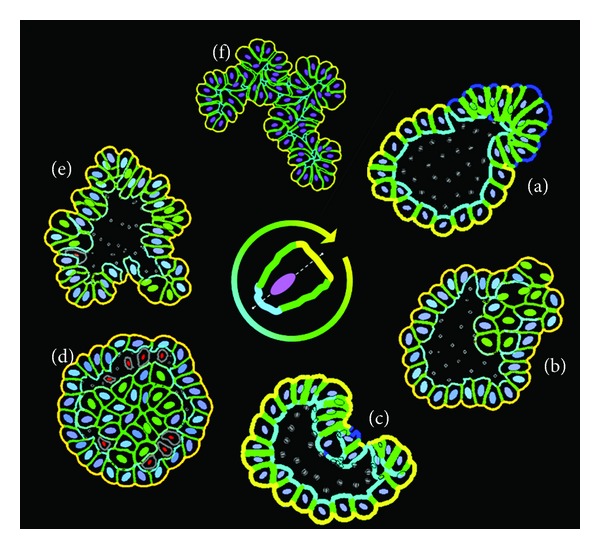
Irregular multicellular morphologies arising after disruption of certain epithelialpolarization signals. Upon dysregulation of one of more of basal, lateral, or apical signals, an individual cell may lose its ability to properly polarize that in turn may result in the emergence of irregular acinar morphologies. The altered signals are identified by a color on the inner circle: yellow-basal (ECM) signals; cyan-apical signals; green-lateral signals. The center picture shows schematics of a polarized cell with typical distribution of cell membrane receptors.

During the development of epithelial tissues, the outer (basal) cells secrete various proteins, such as collagens and laminins, that accumulate in the form of a stiff supportive basement membrane (BM) surrounding the whole structure [15]. Epithelial cells are capable of attaching to the basement membrane through the special cell-ECM transmembrane receptors, such as integrins. In *IBCell,* the formation of BM is modeled by accumulating ECM proteins and changing the role of cell receptors from *growth* to *basal* (ECM) receptors (Figure 2 and the appendix). However, when the ECM receptors in the host cell are downregulated, their role is changed back to the default state: *growth*. Thus, the ratio of growth receptors increases, and the cell is able to reenter the proliferation process. Since all other signals (lateral and apical) are assumed to be unchanged, the growing cells divide in a typical epithelial fashion, that is, orthogonal to the lumen. It has been reported in [19] that normal epithelial cells acquire two different orientations of cell division: either orthogonal or parallel to the lumen. The orthogonal division along the basal-apical axis results in two luminally positioned daughter cells and leads to duct expansion. In our simulation, the elevated cell proliferation accompanied with basal-apical orientation of cell division axis results in the emergence of a new tubular duct sprout shown in Figure 3(a). This is consistent with experiments showing initiation and outgrowth of cell cohorts forming new tubule-like structures in the three-dimensional cultures of Madin-Darby canine kidney (MDCK) epithelial cells [20]. 

Immersed Boundary Equations.

(1) ρ(∂u(x,t)∂t+(u(x,t)·∇)u(x,t)) =−∇p(x,t)+μΔu(x,t)+μ3ρ∇s(x,t)+f(x,t),

(2) $$\begin{array}{c} \rho \nabla \cdot u\left(x,t\right)=s\left(x,t\right), \end{array}$$

(3) $$\begin{array}{c} f\left(x,t\right)=\int_{\Gamma }^{}F\left(l,t\right)\delta \left(x-X\left(l,t\right)\right)dl \end{array}$$

(4) $$\begin{array}{c} F\left(l,t\right)={ℱ}_{*}\frac{||X\left(l_{*},t\right)-X\left(l,t\right)||-{ℒ}_{*}}{||X\left(l_{*},t\right)-X\left(l,t\right)||}\left(X\left(l_{*},t\right)-X\left(l,t\right)\right), \end{array}$$

(5) s(x,t)=∑k∈Ξ+S+(Yk,t)δ(x,Yk)+∑k∈Ξ−S−(Zm,t)δ(x,Zm),

(6) $$\begin{array}{c} \frac{\partial X\left(l,t\right)}{\partial t}=u\left(X\left(l,t\right),t\right)=\int_{\Omega }^{}u\left(x,t\right)\delta \left(x-X\left(l,t\right)\right)dx, \end{array}$$

(7) $$\begin{array}{c} \frac{\partial \gamma \left(X\left(l,t\right)\right)}{\partial t}=\kappa_{1}X\left(l,t\right)-\kappa_{2}\gamma \left(X\left(l,t\right)\right). \end{array}$$

### 2.3. Alterations in ECM and Apical Signals

We have previously hypothesized [21] that disruption in both cell-ECM adhesion signals and apical signals results in the loss of tissue homeostatic balance and initiation of cell hyperplastic growth, leading to both luminal filling and invasion of the surrounding tissue (Figure 3(b)). If such changes are only present locally in one epithelial cell, the remaining cells can still form a functional duct. However, if more cells are affected, the overall cluster morphology resembles more an unorganized multicellular spheroid. Similar results have been shown experimentally when the ECM stiffness was modified that resulted in alterations of ECM signaling and in the loss of cell epithelial polarity leading to growth of aberrant multicellular structures [22]. In contrast, when certain oncogenes were overexpressed in individual cells growing within the organotypic mammary acini, the mutated cells were able to initiate luminal outgrowth without disrupting the ductal structure [23].

### 2.4. Alterations in Apical Signals

We used *IBCell* to simulate a case when a randomly chosen single cell loses its apical markers. This resulted in an elevated ratio of growth receptors leading to reinitiation of cell proliferation process. Since all other signals (lateral and basal) were unchanged, the host cell was able to maintain its basal and lateral membrane domains. As a result, all growing cells were dividing in a typical epithelial fashion (orthogonal to the lumen), and the duct integrity was preserved. However, the elevated cellular proliferation with preserved orientation of cell division led together to bending of the duct towards the side with abnormal apical signals producing a concaved cleft (Figure 3(c)). Prolonged growth would lead to the closure of the luminal space and to the collapse of the whole duct. Whether similar processes can occur in epithelial ducts in the living organisms has not been documented. However, the process of cleft formation has been observed, for example, during salivary glands morphogenesis, although not in the hollow ducts but in the glands fully filled with cells [24].

### 2.5. Alterations in Apical and Death Signals

Normal development of epithelial cysts requires clearance of the luminal space from all inner cells. Different processes could be responsible for this selective cell removal, however, it has been shown experimentally that cell apoptotic death is the necessary contributor to the formation and maintenance of the empty luminal space [25]. This form of cell death is induced on purpose and often called *programmed cell death*. However, it is not known how the process of lumen clearance is triggered. We previously used *IBCell* to test the hypothesis that apoptotic death arises in cells directly attached to the polarized cells and is initiated upon the formation of the apical membrane domain and disassembly of the adhesive links between polarized and inner cells [11]. Here, we simulate an opposite scenario. When cell response to apical signal is altered in such a way that cell apoptotic death in adjacent cells is not initiated, these cells can sense free space in their vicinity and reinitiate the growth process. As a result, the inner lumen space can be repopulated (Figure 3(d)). This kind of escape from tissue homeostatic balance leads to the formation of mutants resembling ductal carcinoma *in situ*, a noninvasive form of ductal tumors characterized by intact overall ductal structure with filled luminal space [7].

### 2.6. Alterations in Lateral but Not Basal and Apical Signals 

We previously showed in [11] that in order to form round regular ductal structures, the orientation of mitotic spindle poles and the axis of cell division need to be coordinated in *IBCell*. The expansion of the epithelial layer of cells requires symmetrical cell division (along the apical-basal axis) resulting in two luminally positioned daughter cells. In contrast, asymmetrical cell division (parallel to the basement membrane and/or lumen) gives rise to one basally and one luminally positioned daughter cell and culminates in either cell differentiation or its apoptosis. As a result, the dying cell contributes to lumen expansion. The rule of choosing one of the directions was based on cell shape, with the asymmetrical direction chosen when the cell was elongated along the basal-apical axis. Cell elongation might be due, for example, to the pressure of tightly packed neighboring cells or may arise from cell-cell adhesion forces. Here, we simulated the case when epithelial cells in the duct gained the proliferative phenotype due to disruption in cell-cell lateral signaling followed by acquiring additional growth receptors. However, we assumed that the basal and apical signals are not altered, so the growing cells will divide symmetrically. This leads to increased lateral tension and results in duct bending (Figure 3(e)), because an excess of epithelial cells is not accompanied by the expansion of the lumen inside the duct. 

### 2.7. Alterations in Lateral and Apical Signals

Our final simulation (Figure 3(f)) tests a scenario when all cell divisions in the developing structure are symmetrical, that is, orthogonal to the basal membrane domain. In this case, all cells have minuscule apical sides and almost no luminal space. Since the lateral signals are altered, the cells have excess of growth receptors and proliferate continuously leading to the formation of highly irregular multicellular structure with no detectable lumen. The importance of the orientation of cell division axis was investigated experimentally in 3D cultures of MDCK cells [26], where it was shown that if the angle between apico-basal axis and the spindle axis (which is perpendicular to the axis of cell division) is less than 45°, the formation of inner lumen is suppressed. 

## 3. Discussion

Maintaining the structural homeostatic balance in tissues is essential for their proper function. Certain changes in tissue architecture do take place even in an adult healthy organism, but there are constrains in terms of the amount of disruption the cells and tissues can withstand, and how long they are exposed to the disruption clues. For example, it has been determined, based on morphological identification of both mitotic and apoptotic events [27], that cell turnover in lobules of the normal human breast undergoes significant cyclical changes during the menstrual cycle, with the peak for apoptosis occurring 3 days after the peak for mitosis. An even more spectacular example of a controlled tissue structure remodeling is the process of involution—a programmed destruction and removal of the breast epithelial ducts that were generated during pregnancy to enable milk production. This postlactation regression involves a massive death of epithelial cells and remodeling of epithelial ducts to their prepregnant state and function [28]. However, uncontrolled homeostatic imbalance may lead to nonreversible changes in tissue microarchitecture and subsequently to its malignancy. 

Cancer is a genetic disease, but the mutated cell manifests its tumorous characteristics by changing its physical behavior. For instance, the invasive cell must overcome adhesive forces exerted by the neighboring cells, apply repulsive forces to break through the basement membrane surrounding the epithelial duct, and utilize pulling and pushing forces to actively move through the extracellular matrix. Thus, in addition to indispensible functions of genetics programs, all cells and tissues are also shaped by mechanical forces and stresses. Tumor progression is characterized by an incremental stiffening of the tissue. In fact, breast self-exam, the preliminary cancer screening method, looks for unusual differences in tissue density and stiffness, such as lumps or nodules.

On the other hand, homeostatic mechanisms are built using the cells' existing molecular and signaling machinery as well as the architecture of the tissue and other physical constraints. Despite their general robustness against the most common perturbations, some genetic mutations and certain changes in tissue architecture could disrupt homeostasis. Moreover, there is increasing experimental evidence showing that the restoration of tissue organization is able to repress the malignant phenotype of genetically aberrant cells. For example, when mouse embryonal carcinoma cells (which form malignant tumors upon subcutaneous injection) were fused with normal blastocysts, they were able to give rise to phenotypically normal cancer-free mouse [29]. Also, malignant T4-2 cells forming disorganized continuously proliferating colonies can be reverted to near-normal phenotype when grown in the presence of integrin-blocking antibodies. These reverted T4-2 cells formed regular growth-arrested acinar structures with restored apico-basal polarity, reorganized actin cytoskeleton, and were able to remain quiescent for up to 1 month in culture [30]. 

We have shown using the biomechanical model of epithelial ducts, *IBCell*, that alterations in cell intrinsic responses to extrinsic signals lead to the loss of tissue integrity if such microenvironmental perturbations are exerted in a persistent and/or prolonged way. When cell epithelial character is disturbed by changes in the expression of certain cell membrane receptors or markers, the uncontrolled growth is triggered leading to aberrations in the epithelial duct structure (altered lateral and/or apical signaling), or to the emergence of microinvasions (misread ECM signaling). These examples show that microscopic changes in cell mechanics, extracellular matrix structure, and cell-matrix interactions can dysregulate molecular mechanisms of mechanotransduction. Thus, the physical basis for epithelial cancer initiation might be attributed to either altered force balance on cellular or tissue level or the perturbed cellular responses to various mechanical stimuli.

## Appendix

### Mathematical Model

The *IBCell* was introduced in [31] to model early tumor development and subsequently applied to investigate the sufficient and necessary conditions for the formation and stability of hollow epithelial acini, three-dimensional multicellular culture systems that recapitulate the structure and function of epithelial mammary cysts [10, 11]. In this model, the eukaryotic cell is represented as a two-dimensional fully deformable body, and its structure includes an elastic plasma membrane modeled as a network of linear springs that define cell shape and encloses the viscous incompressible fluid representing the cytoplasm and providing cell mass. These individual cells can interact with other cells and with the environment via a set of discrete membrane receptors located on the cell boundary. In particular, each boundary point can be engaged in adhesion either with one of the neighboring cells or with the extracellular matrix, and cell membrane receptors can be used to sense the presence of other cells or extracellular matrix in the local cell vicinity. The host cell can initiate certain cell-life processes, such as proliferation, division, apoptotic death, or epithelial polarization, based on its membrane receptors signature (a distribution of growth, death, apical, cell-cell, and cell-ECM adhesion receptors). More precisely, cell growth is modeled by placing point sources and sinks around the cell boundary to model transport of fluid through the cell membrane, and, once the cell area is doubled, the contractile ring is formed by introducing springs between opposite points on the cell boundary that upon contraction split the cell into two daughters. Cell epithelial polarity is acquired by developing three distinct cell membrane domains: basal, defined by cell membrane receptors contacting the external media; lateral, defined by cell receptors being in contact with other cells; apical, facing the hollow lumen. Cell apoptotic death is modeled by placing point sinks and sources along the membrane of the whole cell to release fluid from the cell interior to the extracellular space. The *IBCell* model is based on the immersed boundary framework and governed by (1)–(7).

In this system, (1) is the Navier-Stokes equation of a viscous incompressible fluid defined on the Cartesian grid *x* = (**x**_1_, **x**_2_), where *p* is the fluid pressure, *μ* is the fluid viscosity, *ρ* is the fluid density, *s* is the local fluid expansion, and *f* is the external force density. Equation (2) is the law of mass balance. Interactions between the fluid and the material points *X*(*l*, *t*), on cell boundaries Γ and at point sources **Y**_**k**_ and sinks **Z**_**m**_ placed in the cell local microenvironment, are defined in (4), (5), (6). Here, the force density **F**(*l*, *t*) defined on cell boundaries, and the sources *S* ^+^(**Y**_**k**_, *t*) and sinks *S* ^−^(**Z**_**m**_, *t*) defined in the cell microenvironment, are applied to the fluid using the two-dimensional Dirac delta function *δ*, while all material boundary points *X*(*l*, *t*) are carried along with the fluid. The boundary forces **F**(*l*, *t*) arise from elastic properties of cell boundaries, from cell-to-cell adhesion, and from contractile forces splitting a cell during its division. The sources *S* ^+^(**Y**_**k**_, *t*) and sinks *S* ^−^(**Z**_**m**_, *t*) are chosen such that they balance around each cell separately. The kinetics of ECM proteins *γ*(*x*, *t*) is defined along the cell boundaries and include: constant secretion (at a rate *k*_1_) along the cells' basal domains and its decay (at a rate *k*_2_) around all cells' boundaries. More details on the mathematical formulation of *IBCell* and the implementation of cell-life processes can be found in [31]. The model is implemented in Fortran with all graphical routines executed using Matlab. A typical simulation of developing acinar structures, like these presented here, lasts up to 6 hours on the one-processor desktop computer. The corresponding *in vitro* experiments take about 24–30 days. The *IBCell *simulations can be found at http://www.rejniak.net/ under research option, and the code may be obtained from the author upon request.
